# Detection of Red Blood Cell Membrane Proteins in Myelodysplastic Syndromes Using Eosin-5-Maleimide (EMA) Staining by Flow Cytometry

**DOI:** 10.3390/hematolrep14010003

**Published:** 2022-02-28

**Authors:** Navavee Uman, Sirorat Kobbuaklee, Patsita Kansuwan, Phandee Watanaboonyongcharoen, Chantana Polprasert

**Affiliations:** 1 Department of Medicine, Faculty of Medicine, Chulalongkorn University and King Chulalongkorn Memorial Hospital, Bangkok 10230, Thailand; navavee@outlook.com (N.U.); siroratsk@hotmail.com (S.K.); patsitacu@gmail.com (P.K.); 2 Research Unit in Translational Hematology, Chulalongkorn University, Bangkok 10230, Thailand; 3 Department of Clinical Pathology, Faculty of Medicine, Chulalongkorn University and King Chulalongkorn Memorial Hospital, Bangkok 10230, Thailand; phandee_lee@yahoo.com

**Keywords:** red blood cell membrane disorders, flow cytometry, Eosin-5-Maleimide (EMA) staining, Hereditary Spherocytosis (HS), Southeast Asian Ovalocytosis (SAO), Myelodysplastic syndromes (MDS)

## Abstract

Background: Eosin-5-Maleimide (EMA)-based flow cytometry binds to red blood cell (RBC) membrane-associated proteins which can be used to detect red blood cell (RBC) membrane disorders. Myelodysplastic syndromes (MDS) are stem cell disorders resulting in ineffective hematopoiesis which is commonly present with anemia and erythroid dysplasia. Objectives: We aimed to study RBC membrane defects in MDS using flow cytometry for EMA staining. Methods: We enrolled anemic patients who were diagnosed with low-risk MDS (R-IPSS score ≤ 3.5), RBC membrane disorders [hereditary spherocytosis (HS) and Southeast Asian ovalocytosis (SAO)], and normal controls. Complete blood count (CBC) and flow cytometry for EMA staining were performed. Results: There were 16 cases of low-risk MDS, 6 cases of RBC membrane disorders, and 15 control cases. Mean fluorescence intensity (MFI) of EMA binding test in the RBC membrane disorders was significantly lower than controls (17.6 vs. 24.3, *p* < 0.001), but the EMA binding test in the low-risk MDS was not significantly different than the controls (26.5 vs. 24.3, *p* = 0.08). Conclusion: the RBC membrane defect in low-risk MDS was not demonstrated as having detection ability using EMA binding test with flow cytometry.

## 1. Introduction

Myelodysplastic syndromes (MDS) are a group of clonal bone marrow neoplasms with variable disease progression. Approximately 50% of MDS patients present with anemia and a hemoglobin of less than 10 g/dL [1]. Anemia in low-risk MDS is mainly caused by ineffective hematopoiesis resulting in dysplastic morphology and excessive apoptosis of erythroblasts [2]. Dyserythropoiesis in MDS is characterized by an abnormal terminal differentiation of erythroblasts. The proportion of different stages of erythroblasts can be detected by dynamic changes in the expression of membrane protein [3]. Moreover, non-immune hemolysis of unknown pathophysiology has been reported as 10% in MDS [4]. Therefore, abnormal of red cell membrane proteins in MDS should be explored. Red blood cell (RBC) membrane defect is one of the factors causing anemia in RBC membrane disorders such as: hereditary spherocytosis (HS) and Southeast Asian ovalocytosis (SAO). Hereditary spherocytosis is caused by germline mutation in genes coding RBC cytoskeleton proteins such as band 3, spectrin, ankyrin and protein 4.2. These mutations cause a disruption in the RBC structure and manifest hemolytic anemia [5]. SAO is caused by a heterozygous 27-nucleotide deletion in *SLC4A1* coding for band 3, the anion exchange protein of the red cell membrane [6]. Eosin-5-maleimide (EMA) is a fluorescent dye which binds to epsilon-NH_2_ group of lysine on band 3 and Rh-related proteins on red blood cells [7,8]. Therefore, the detection of EMA by flow cytometry can be used for making a diagnosis of hereditary spherocytosis (HS) and other red cell membrane disorders. In this study, we aimed to evaluate red blood cell membrane defects in patients with low-risk myelodysplastic syndromes who predominately presented with anemia. Eosin-5-Maleimide staining by flow cytometry was used to compare those with RBC membrane disorders and a control group.

## 2. Materials and Methods

We collected 3 mL of blood specimens from consecutive patients with low-risk MDS, HS, SAO and controls at Hematology Clinic, King Chulalongkorn Memorial Hospital. All of the subjects were older than 18 years. For the low-risk MDS, we recruited patients diagnosed with MDS according to the WHO classification criteria of 2016 [9] with an R-IPSS ≤3.5. The RBC membrane disorders were diagnosed by both clinical and laboratory investigation of chronic non-immune hemolysis and red cell morphology (presence of spherocytes in HS and macro-ovalocytes in SAO). For the control group, we recruited healthy volunteers with no history or family history of hematologic disease, and hemoglobin levels greater than 11.5 g/dL in women and 13.0 g/dL in men.

Complete blood count (CBC) and flow cytometry using Eosin-5-Maleimide (EMA) staining adapted from a previous study, [10] was performed. In brief, each blood specimen was added to a tube with phosphate buffered saline (PBS) with pH of 7.5 and then washed 3 times. The washed red cells were incubated in 25 µL of 0.5 mg/mL EMA staining (FITC, Biotium, Catalog#92013) with intermittent mixing in a capped plastic 0.5-mL microtube in the dark at room temperature for 1 h. After the unbound dye supernatant was removed and washed 3 times, 1 mL of PBS was added. Flow cytometric analysis using BD FACSCanto II flow cytometer and 15,000 RBC events were acquired. After the acquisition of scattergrams for forward scatter (FSC) and side scatter (SSC), RBCs with high FSC and SSC were gated, and mean fluorescence intensity (MFI) of EMA was measured in each subject. We used Kaluza version 1.2 to analyze the data. This study was approved by the Institutional Review Board and the Ethics Committee of the Faculty of Medicine, Chulalongkorn University.

### Statistical Analysis

Data analysis was performed using Stata version 15.1 (Stata Corp., College Station, TX, USA). For descriptive analysis, the frequencies and percentage of categorical variables were calculated, while mean, standard deviation (SD), median, percentile 2.5th and percentile 97.5th were calculated for continuous variables. The Wilcoxon rank-sum (Mann-Whitney U) test or the two-sample independent T-test were used to compare medians or means between two groups. Chi-square or Fisher’s exact test were used to compare proportions for categorical data. Linear regression was used for unadjusted and adjusted mean differences of EMA between groups. All of the *p*-values reported were 2-sided and statistical significance was defined as *p* < 0.05.

## 3. Results

We examined 16 cases of low-risk MDS presenting with anemia, 6 cases of RBC membrane disorders (5 cases of HS, 1 case of SAO), and 15 control cases. The baseline characteristics and red blood cell index from the complete blood count (CBC) of the study population are presented in Table 1. Median age of the patients in the RBC disorder, low-risk MDS and control groups were 36, 76 and 28 years old, respectively. The mean hemoglobin levels in the RBC membrane disorder, low-risk MDS and control groups were 10.6, 8.6 and 13 g/dL, respectively, (*p* < 0.001). The mean corpuscular hemoglobin concentration (MCHC) in the RBC membrane disorder group was significantly higher than the low-risk MDS groups (34.2 ± 1.4 vs. 31.9 ± 1.3, *p* < 0.001). Red blood cell distribution width of the RBC membrane disorder group was significantly higher than the control group (18.6 ± 4.3 vs. 12.8 ± 0.7, *p* < 0.001).

**Figure 1:**
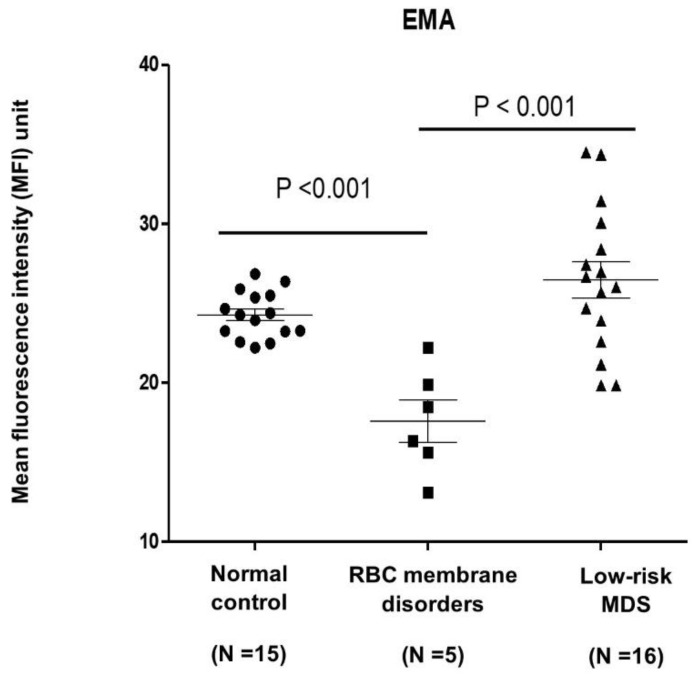
Comparison of EMA binding test (mean fluorescent intensity, MFI) using flow cytometry between groups. EMA binding test of RBC membrane disorders was lower than low-risk MDS (*p* < 0.001) and normal control (*p* < 0.001) by independent two sample *t*-test, but the expression EMA between low-risk MDS and normal control did not differ.

EMA binding test using flow cytometry by Eosin-5-Maleimide (EMA) staining in the RBC membrane disorders group (N = 6) was significantly lower than the controls (N = 15) (17.6 vs. 24.3, *p* < 0.001). EMA binding test in the low-risk MDS did not show a difference compared to the controls (26.5 vs. 24.3, *p* = 0.08) (Figure 1). When adjusted by age, gender and MCV using a linear regression model, the mean difference of EMA binding test in the RBC membrane disorder group was 6.9-fold lower than in the controls (*p* < 0.001). No difference between the low-risk MDS and the normal control group was observed (Table 2).

In the RBC membrane disorders group (*n* = 6), 5 cases were HS and 1 case was SAO. Hemoglobin, MCHC and RDW in the HS were higher than the SAO (11 ± 3.1 vs. 7 g/dL, 34.4 ± 1.6 vs. 33 g/dL and 19.5 ± 4.1 vs. 14%, respectively). MCV and MCH in the HS were lower than the SAO (84.3 ± 7.1 vs. 90 fL and 29.2 ± 2.6 vs. 32 pg). EMA binding test in HS and SAO was 18.01 ± 3.5 and 15.62, respectively (Table 3).

## 4. Discussion

Pathogenesis of anemia in MDS is linked to ineffective erythropoiesis. Impaired terminal erythroid differentiation has been demonstrated in MDS [3] and has been shown to be a prognostic marker for survival in MDS [11]. In our study, the RBC membrane defect in low-risk MDS was not detected by EMA binding using flow cytometry. This suggests that the dysplastic morphology of red blood cells does not relate to abnormal cytoskeleton proteins of the red blood cells.

EMA staining is a standard method for detecting RBC membrane defect. In this study, we demonstrated RBC membrane defect by using EMA staining method in the RBC membrane disorder group. The expression of EMA is affected by age, sex and MCV [12]. Thalassemia trait which causes low MCV is common in Thailand. Previous studies recommended comparing EMA binding with age-matched samples [13]. In this study, the control group was younger than the low-risk MDS (median age: 28 vs. 76 years old), which is a limitation of our results. Although the number of patients and controls were small, we were able to demonstrate a difference between the EMA binding test in the RBC membrane disorder group and the controls.

## 5. Conclusions

The EMA binding test using flow cytometry method is a useful tool in making a diagnosis of RBC membrane disorder, especially HS. The EMA binding test in the low-risk MDS group was not different from the controls which suggested that RBC membrane defect was not the primary cause of anemia in the low-risk MDS group.

## Figures and Tables

**Table 1:** Baseline characteristics and red blood cell index values.

	Control (*N* = 16)	MDS (*N* = 15)	RBC Membrane Disorders (*N* = 6)	*p*-Value
Median (IQR) age (years)	28 (27–30)	76 (67–82)	36 (27–45)	<0.001
Gender, N (%)				0.07
Female	11 (73.3)	5 (31.3)	4(66.7)	
Male	4 (26.7)	11 (68.7)	2 (33.3)	
Hemoglobin (g/dL)	13 (1.0)	8.6 (1.8)	10.3 (3.2)	<0.001
MCV (fL)	86.2 (5.7)	92.5 (17.1)	85.2 (6.8)	0.62
MCH (pg)	28.7 (2.3)	29.5 (5.6)	29.6 (2.6)	0.77
MCHC (g/dl)	33.2 (0.7)	31.9 (1.3)	34.2 (1.4)	<0.001
RDW (%)	12.8 (0.7)	18.5 (3.9)	18.6 (4.3)	<0.001

MCV = mean corpuscular volume, MCH = Mean corpuscular hemoglobin, MCHC = mean corpuscular hemoglobin concentration, RDW = red blood cell distribution width PLT = platelet, WBC = white blood cell.

**Table 2:** Unadjusted and adjusted mean different of EMA between MDS and HS compared with normal group using linear regression model.

	Unadjusted		Adjusted *	
	Mean Difference (95%CI)	*p*-Value	Mean Difference (95%CI)	*p*-Value
Group				
Normal	Ref.	Ref.	Ref.	Ref.
MDS	2.2 (−0.18 to 4.58)	0.08	1.5 (−6.07 to 9.17)	0.68
HS	−6.7 (−10.03 to −3.34)	<0.001	−6.87 (−10.48 to −3.26)	0.001

* Adjusted by age, sex and MCV.

**Table 3:** Characteristics of the RBC membrane disorders group.

ณNo.	1	2	3	4	5	Mean (SD)	6
Group	HS	HS	HS	HS	HS	-	SAO
Age	26	47	27	32	40	34.4 (8.9)	45
Gender	Female	Female	Female	Male	Male	-	Female
Hb (g/dL)	9.2	10.4	10.2	8.8	16.4	11 (3.1)	7
MCV (fL)	81	87.6	73.3	90.4	89	84.3 (7.1)	90
MCH (pg)	27.8	29.7	25.7	29.9	32.7	29.2 (2.6)	32
MCHC (g/dl)	33.1	33.9	35.1	33	36.8	34.4 (1.6)	33
RDW (%)	21.1	19.6	23.4	20.7	12.5	19.5 (4.1)	14
PLT	250	237	211	286	328	262 (45.5)	160.2
(×103 cell/mm^3^)							
WBC (cell/mm^3^)	11,300	8320	9820	7010	6590	8608 (1.9)	9600
EMA	22.23	16.35	18.48	19.88	13.09	18.01 (3.5)	15.62

SAO = Southeast Asian Ovalocytosis, HS = Hereditary Spherocytosis, MCV = mean corpuscular volume, MCH = Mean corpuscular hemoglobin, MCHC = mean corpuscular hemoglobin concentration, RDW = red blood cell distribution width, PLT = platelet, WBC = white blood cell.

## References

[1] Castelli R., Schiavon R., Rossi V., Deliliers G.L. (2018). Management of anemia in low-risk myelodysplastic syndromes treated with erythropoiesis-stimulating agents newer and older agents. Med Oncol..

[2] Cazzola M., Barosi G., Berzuini C., Dacco M., Orlandi E., Stefanelli M., Ascari E. (1982). Quantitative evaluation of erythropoietic activity in dysmyelopoietic syndromes. Br. J. Haematol..

[3] Hu J., Liu J., Xue F., Halverson G., Reid M., Guo A., Chen L., Raza A., Galili N., Jaffray J. (2013). Isolation and functional characterization of human erythroblasts at distinct stages: Implications for understanding of normal and disordered erythropoiesis in vivo. Blood.

[4] Komrokji R.S., Al Ali M.N., Hussaini M.O., Sallman D.A., Rollison D.E., Padron E. (2020). U2AF1 and EZH2 Mutations Are Associated with Non-Immune Hemolytic Anemia in Myelodysplastic Syndromes. Blood.

[5] Glenthøj A., Sharfo A., Brieghel C., Nardo-Marino A., Birgens H., Petersen J.B. (2020). Improving the EMA Binding Test by Using Commercially Available Fluorescent Beads. Front. Physiol..

[6] Picard V., Proust A., Eveillard M., Flatt J.F., Couec M.-L., Caillaux G., Feneant-Thibault M., Finkelstein A., Raphael M., Delaunay J. (2014). Homozygous Southeast Asian ovalocytosis is a severe dyserythropoietic anemia associated with distal renal tubular acidosis. Blood.

[7] King M.-J., Smythe J.S., Mushens R. (2004). Eosin-5-maleimide binding to band 3 and Rh-related proteins forms the basis of a screening test for hereditary spherocytosis. Br. J. Haematol..

[8] Adan A., Alizada G., Kiraz Y., Baran Y., Nalbant A. (2016). Flow cytometry: Basic principles and applications. Crit. Rev. Biotechnol..

[9] Arber D.A., Orazi A., Hasserjian R., Thiele J., Borowitz M.J., Le Beau M.M., Bloomfield C.D., Cazzola M., Vardiman J.W. (2016). The 2016 revision to the World Health Organization classification of myeloid neoplasms and acute leukemia. Blood.

[10] Park S.H., Park C.-J., Lee B.-R., Cho Y.-U., Jang S., Kim N., Koh K.-N., Im H.-J., Seo J.-J., Park E.S. (2014). Comparison Study of the Eosin-5′-Maleimide Binding Test, Flow Cytometric Osmotic Fragility Test, and Cryohemolysis Test in the Diagnosis of Hereditary Spherocytosis. Am. J. Clin. Pathol..

[11] Ali A.M., Huang Y., Pinheiro R.F., Xue F., Hu J., Iverson N., Hoehn D., Coutinho D., Kayani J., Chernak B. (2018). Severely impaired terminal erythroid differentiation as an independent prognostic marker in myelodysplastic syndromes. Blood Adv..

[12] Ciepiela O., Adamowicz-Salach A., Bystrzycka W., Łukasik J., Kotuła I. (2015). Mean corpuscular volume of control red blood cells determines the interpretation of eosin-5′-maleimide (EMA) test result in infants aged less than 6 months. Ann. Hematol..

[13] Falay M., Ulusan G.E., Şenes M., Acar İ.O. (2018). Are the Reference Ranges and Cutoff Values of Eosin-5′-Maleimide (EMA) Binding Test for Hereditary Spherocytosis Specific for Each Age Group?. Clin. Lab..

